# Superabsorbent Hydrogels Derived from Unpurified Sargassum Biomass via Direct Carboxymethylation and Crosslinking

**DOI:** 10.3390/gels12050431

**Published:** 2026-05-15

**Authors:** Cleny Villalva-Cañavi, Alma Berenice Jasso-Salcedo, Daniel Lardizabal-Gutierrez

**Affiliations:** 1 Centro de Investigación en Materiales Avanzados (CIMAV), Miguel de Cervantes 120, Chihuahua 31136, Mexico; cleny.villalva@cimav.edu.mx (C.V.-C.); alma.jasso@cimav.edu.mx (A.B.J.-S.); 2 Investigadoras e Investigadores por México, Secretaría de Ciencia, Humanidades, Tecnología e Innovación (SECIHTI), Av. Insurgentes sur 1562, Col. Crédito Constructor, Alcaldía Benito Juárez, Ciudad de México 03940, Mexico

**Keywords:** hydrogels, CMC, sargassum, superabsorbents, algae, environmental remediation

## Abstract

The atypical proliferation of Sargassum (*Sargassum* spp.) in the tropical Atlantic and the Caribbean Sea over the past decade has triggered an unprecedented environmental and socioeconomic crisis along the Mexican coastline. Continuous beaching events of this macroalga on the Riviera Maya have caused coastal ecosystem degradation, severe impacts on the tourism sector, toxic gas emissions during decomposition, and high cleanup costs. To address this challenge, the valorization of Sargassum as a raw material for synthesizing functional materials represents a sustainable management strategy. In this study, a superabsorbent hydrogel was developed from Sargassum biomass (collected in Cancún, Quintana Roo, in 2025) using an innovative process that bypasses the conventional cellulose isolation step. The biomass was subjected to high-energy milling (15 and 30 min) to prepare Sargassum powder, which was subsequently carboxymethylated using monochloroacetic acid. This modified biomass was then crosslinked with citric acid, a process evaluated at three different citric acid/carboxymethylated Sargassum mass ratios. The hydrogel synthesized with the lowest crosslinking agent ratio achieved a maximum water absorption capacity of 1160 wt%, a value that exceeds the typical absorption capacities of 700–900% for biopolymer hydrogels. Successful material formation was confirmed by Fourier transform infrared spectroscopy (FTIR), which revealed the characteristic functional groups of CMC and the ester bonds formed during crosslinking. Additionally, scanning electron microscopy (SEM) analysis showed a well-defined porous structure with pore sizes ranging from 8.5 to 19.5 µm, which is essential for its high absorption performance. This study demonstrates the feasibility of producing high performance hydrogels from Sargassum through a simplified, cost-effective, and environmentally friendly process. These findings open a promising avenue for the integrated management of this problematic biomass, transforming it into value-added materials with potential applications in agriculture, hygiene, and environmental remediation.

## 1. Introduction

Over the past decade, the coasts of the Mexican Caribbean have faced an unprecedented ecological crisis due to massive macroalgal stranding [1]. This phenomenon began to be widely documented between 2014 and 2015, with biomass accumulations reaching up to 2360 m^3^ km^−1^. However, the issue reached critical levels during the 2018–2019 period, with deposits of up to 20 kg m^−2^ (wet weight) recorded during peak influx months [2]. During the high season (May–September), Riviera Maya beaches receive between 5000 and 10,000 metric tons of biomass daily, generating cleanup costs exceeding USD 17 million annually in Quintana Roo alone. Furthermore, these events cause severe impacts on water quality, coral reef mortality due to light attenuation and anoxia, hydrogen sulfide (H_2_S) release during decomposition, and direct disruption of the tourism industry, which serves as the economic backbone of the region [3]. Sargassum is a brown macroalga, primarily represented in the Atlantic Ocean, Gulf of Mexico, and Caribbean Sea by the pelagic species *Sargassum natans* (morphotypes I and VIII) and *Sargassum fluitans* III [4]. Its cellular architecture is best described as a complex polysaccharide matrix dominated by alginates and sulfated polysaccharides (particularly fucoidans), with a minor fibrillar cellulose network embedded [5]. The chemical composition of this biomass is heterogeneous and varies according to geographic origin, seasonality, and the anatomical section analyzed [6]. On a dry weight basis, Sargassum has been reported to contain approximately 38% ash, 15% protein, and a low average lipid fraction of 2.2% [7]. However, its biotechnological value lies in its high carbohydrate content, which accounts for roughly 44% of its dry weight. This carbohydrate fraction consists of storage and structural polysaccharides such as laminarin, mannitol, and cellulose, with a notable presence of fucose-rich alginates and fucoidans [8,9]. The chemical modification of naturally biocompatible and biodegradable algal polysaccharides is a key strategy to optimize their mechanical properties, water retention capacity, and tunable functionality [10]. Among these methods, carboxymethylation stands out as one of the most effective techniques for producing derivatives with superior physical and biological characteristics [11]. Hydrogels are defined as three-dimensional polymeric networks with high hydrophilicity and a crosslinked structure, whose functional versatility enables their implementation in strategic sectors ranging from biomedicine, specifically in controlled drug delivery, tissue engineering, and wound care to advanced applications in the agrochemical, food, and cosmetic industries [12,13]. In the recent literature, hydrogel synthesis from Sargassum has been reported through fractionation of its components; a prominent example is the production of purified alginate matrices ionically crosslinked with CaCl_2_ [14]. This gelation process requires a two-stage kinetics extending over more than 300 h, integrating acid diffusion mechanisms and ionic crosslinking with 0.5 M CaCl_2_ to consolidate the polymer network. Currently, alginate extraction is carried out using the conventional alkaline method with sodium carbonate, or through advanced techniques assisted by microwaves, ultrasound, or enzymes, which significantly improve the yield and purity of the biopolymer. From these extracts, hydrogels reinforced with sargassum-derived nanocellulose obtained via sequential acid and alkaline hydrolysis, have been formulated. The structural stability of these systems is ensured through borax crosslinking, which facilitates the loading and controlled release of nutrients for agricultural applications [8]. In parallel, certain protocols incorporate a pretreatment with formaldehyde and HCl, these derivatives also enable the fabrication of microfilms through the incorporation of collagen and glycerol as a plasticizer [15]. Additionally, high-temperature aqueous extraction (90 °C) has proven to be an effective alternative for isolating high-quality alginates that, when blended with carboxymethyl cellulose (CMC) and subjected to ionic crosslinking, yield highly stable polymeric matrices [16]. As demonstrated in this study, bypassing the conventional extraction and purification of alginate from Sargassum significantly reduces energy consumption and operational costs. To capitalize on this advantage, we propose a streamlined route for synthesizing superabsorbent hydrogels directly from raw biomass. The process integrates high-energy milling with in situ etherification, followed by chemical crosslinking using citric acid as a low-toxicity agent, thereby eliminating the need for traditional isolation of alginate and cellulose fractions. The resulting hydrogels were comprehensively characterized via Fourier transform infrared spectroscopy (FTIR), X-ray diffraction (XRD), thermal analysis, and rheological testing to confirm their chemical structure, crystallinity, and thermal stability. This work establishes a scalable and economically viable pathway for Sargassum valorization, aligned with circular economy principles and offering promising applications in agriculture under water-stress conditions.

## 2. Results and Discussion

### 2.1. Stage 1: “Characterization of Raw and Milled Sargassum”

**Figure 1:**
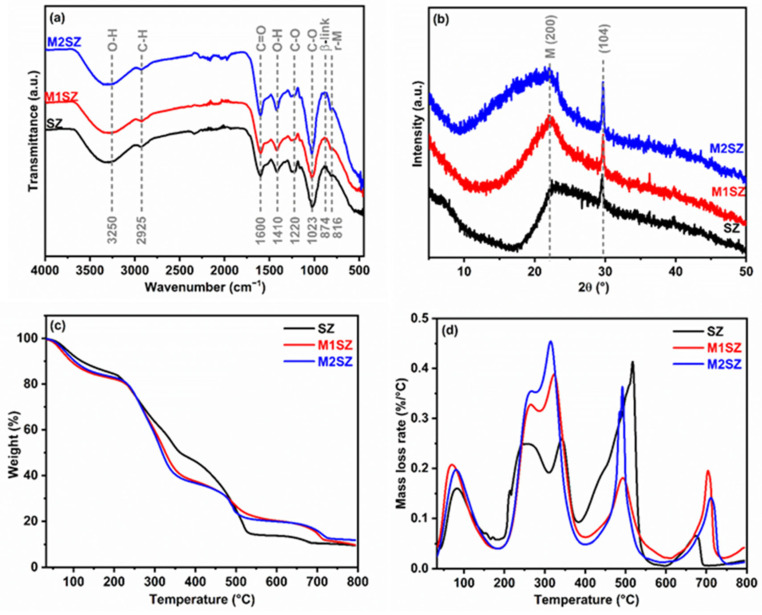
Characterization of Sargassum before and after the mechanochemical activation. (**a**) FTIR spectra, (**b**) XRD patterns (**c**) thermogravimetric analysis (TGA), and (**d**) derivative thermogravimetric (DTG) curves.

Figure 1a shows the FTIR-ATR spectra of untreated Sargassum (SZ) and samples subjected to high-energy milling for 15 min (M1SZ) and 30 min (M2SZ). In the SZ spectrum, a broad band centered at 3250 cm^−1^ is observed, attributed to the O–H stretching vibration, characteristic of polysaccharides [17]. The band observed at 2925 cm^−1^ is assigned to the asymmetric stretching vibrations of C–H bonds in methylene groups (–CH_2_–) present in the backbone of the sugar rings (e.g., the C6 position in glucose units), which confirms the integrity of the characteristic aliphatic backbone of the polysaccharide structure [18]. In the spectra, sharp and intense bands at 1600 cm^−1^ and 1410 cm^−1^ correspond to the asymmetric and symmetric stretching vibrations of the carboxylate group (COO^−^), respectively [19]. These vibrations are characteristic of the mannuronate and guluronate moieties present in the alginate structure [14]. Regarding spectral modifications, variations in the –OH band profile could be related to structural rearrangements or partial oxidation processes, possibly associated with the cleavage of β-glycosidic linkages. This interpretation is supported by the relative increase in the C–O stretching band at 1220 cm^−1^ and the slight decrease in intensity of the band corresponding to the glycosidic linkage (~874 cm^−1^) [20]. However, it is important to note that no new peaks or bands were observed in the M1SZ and M2SZ samples compared to SZ. The spectral profiles remain essentially similar regardless of milling time, suggesting that high-energy milling for up to 30 min does not induce drastic chemical modifications in the functional groups of Sargassum. Consequently, the treatment primarily causes physical changes, preserving the integrity of the primary chemical structure. The X-ray diffraction (XRD) patterns of untreated Sargassum (SZ) and milled samples (M1SZ and M2SZ) are shown in Figure 1b. The diffractogram of the original material exhibits a broad band centered at 2θ ≈ 20–22°, characteristic of polysaccharides with a low degree of crystallinity. Given the predominantly amorphous nature of alginate-based materials, the observed signal is more appropriately attributed to semi-ordered domains or short-range order rather than to a well-defined crystalline plane; nevertheless, in addition to this dominant amorphous fraction, a relatively more defined reflection is observed at 2θ ≈ 22°, corresponding to the (200) plane [21], whose intensity suggests the presence of partially ordered domains within the polymeric matrix. This signal may be associated with regions enriched in β-D-mannuronate (M) blocks, which exhibit greater conformational flexibility and lower steric rigidity than guluronate (G) blocks, favoring a certain degree of local organization. Additionally, an intense peak is identified at 2θ ≈ 29–30°, corresponding to the (104) plane of calcite (CaCO_3_) [22], confirming the presence of the mineral fraction in the marine biomass. The angular and intensity stability of this signal after milling indicates that the treatment does not induce phase transformations in the inorganic component.

The thermal behavior of the material, evaluated by thermogravimetric analysis (TGA) and its derivative (DTG) (Figure 1c,d), reveals a multi-stage degradation process characteristic of Sargassum-derived biopolymers, where three main mass loss regions can be distinguished. Initially, the dehydration stage occurs between room temperature and 130–150 °C, attributed to the evaporation of physically adsorbed water and residual moisture trapped in the porous network; notably, in raw Sargassum samples, this region can extend up to 200 °C [23], including the volatilization of light organic compounds. Subsequently, the primary pyrolytic event occurs between 200 and 400 °C, corresponding to the phase of maximum weight loss driven by the depolymerization and decomposition of structural glycosyl units. Given the predominantly alginate-rich composition of Sargassum, this thermal profile primarily reflects the degradation of alginate chains, which undergo backbone cleavage in the temperature range of 200–370 °C. Overlapping contributions from hemicellulose-like polysaccharides (e.g., fucoidans) are also observed in the 170–400 °C range, while the more thermally stable cellulose fraction degrades at higher temperatures, with a maximum at 308 °C. Due to the complex, heterogeneous nature of the biomass, these degradation events partially overlap, resulting in a broad mass-loss region rather than distinct, isolated peaks [24]. The final stage shows a mass loss in the temperature range of 600 to 800 °C, corresponding to the thermal decomposition of the calcium carbonate present, with the concomitant release of carbon dioxide (CO_2_) [25].

### 2.2. Stage 2: CMC Characterization

**Figure 2:**
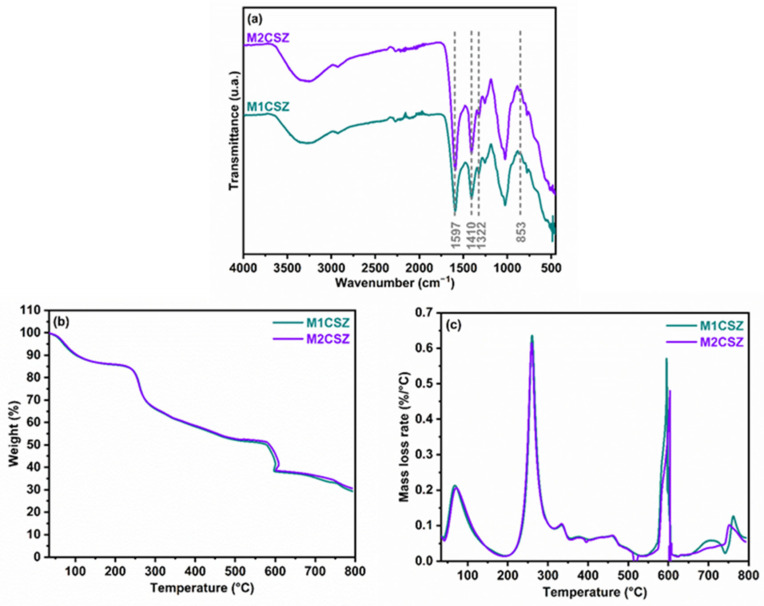
Characterization of carboxymethylated Sargassum. (**a**) FTIR spectra, (**b**) TGA, and (**c**) DTG curves.

Figure 2a presents the FTIR-ATR spectra of carboxymethylated Sargassum evaluated at two milling times, corresponding to samples M1CSZ and M2CSZ. In both spectra, a band around 1597 cm^−1^ is observed, attributed to the asymmetric stretching vibration of the C=O group in –COO^−^, while the peak near 1410 cm^−1^ corresponds to the vibration of the methylene group (–CH_2_–) attached to the carboxyl moiety [26]. Likewise, the signal at 1322 cm^−1^ is associated with the symmetric stretching vibration of the –CO– group, and the peak observed at 853 cm^−1^ indicates that the glycosidic linkage present in the SZ polysaccharide may be associated with specific ring vibrations characteristic of algal polysaccharides. The presence of these characteristic bands in M1CSZ and M2CSZ confirms that the SZ polysaccharide was successfully carboxymethylated at both milling times. Furthermore, no significant spectral differences were observed between the samples, suggesting that increasing the milling time did not produce appreciable structural modifications in the carboxymethylated SZ. The thermogravimetric (TGA) and derivative thermogravimetric (DTG) analyses (Figure 2b,c) of carboxymethylated Sargassum reveal a stepwise decomposition process: first, a mass loss of ~15% due to dehydration (room temperature to 150 °C); this value did not change with the carboxymethylation process, as it remained consistent with that observed for the milled samples. Subsequently, a pronounced weight loss occurs between 200–350 °C due to the degradation of organic compounds such as alginate and hemicellulose; this is followed by a more gradual slope between 350–550 °C associated with the pyrolysis of cellulose [27]; and finally, a sharp drop near 600 °C due to the thermal decomposition of carbonates (CaCO_3_) described above, leaving a final residue of ~30% at 800 °C composed of calcium oxide and ash. The increase in inorganic residue is typically attributed to the incorporation of sodium (Na^+^) from NaOH and sodium monochloroacetate during CMC formation, which generates additional ash (e.g., sodium salts) upon calcination. Comparison between samples M1CSZ and M2CSZ (milled for 15 and 30 min) shows virtually overlapping curves, indicating that the carboxymethylation process yields equivalent conversion regardless of milling time. The DTG analysis shows decomposition maxima at temperatures of 80, 230, and 600 °C, and the complete superposition of the curves is observed in greater detail, inferring highly similar thermal behavior.

### 2.3. Stage 3: Hydrogel Synthesis and Characterization

**Figure 3:**
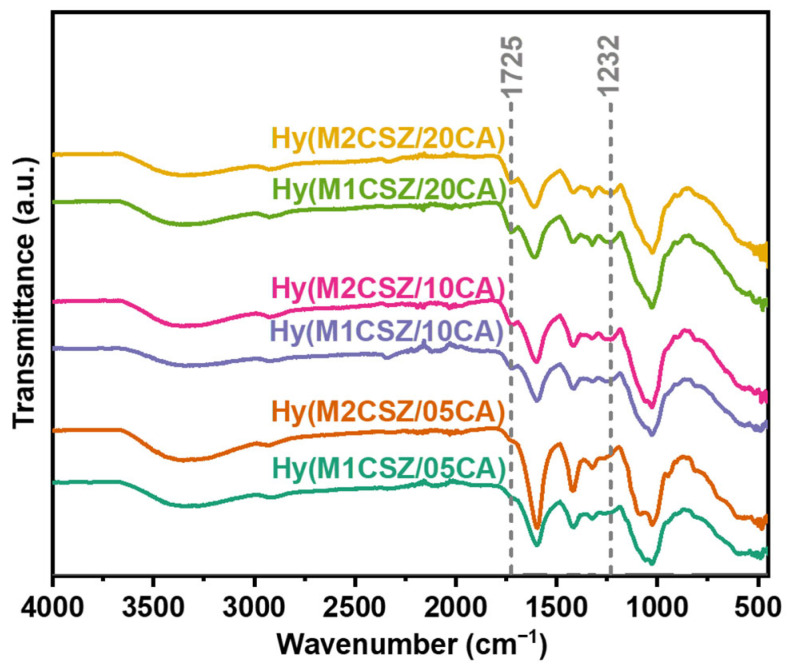
FTIR spectra of the synthesized hydrogels.

FTIR characterization of the synthesized hydrogels, prepared with varying milling times and citric acid concentrations, confirms the preservation of the base polysaccharide structure and the successful formation of the hydrogel network, as evidenced by the consistent spectral profiles and specific diagnostic bands (Figure 3). Characteristic stretching vibrations of O–H (3400–3200 cm^−1^) and C–H (2900 cm^−1^) groups were observed across all samples. Notably, the band at 1725 cm^−1^ (C=O stretching of ester linkages) and the peak at 1232 cm^−1^ (C–O stretching) validate crosslinking via esterification between the polysaccharide matrix and citric acid [28]. The intensity of these bands increases proportionally with citric acid concentration, indicating a higher crosslinking density at the 05CA/CSZ ratio. Spectra obtained at different milling times (15 and 30 min) are virtually superimposable, suggesting that the mechanical treatment does not alter the qualitative chemical composition, although it may enhance the homogeneity of crosslinker distribution. Additionally, the high similarity in the fingerprint region (<1000 cm^−1^) confirms the absence of main chain degradation. A direct correlation is observed between the intensity of the 1725 cm^−1^ peak and network density, where a higher intensity corresponds to a more tightly crosslinked structure and a consequently reduced water absorption capacity.

**Figure 4:**
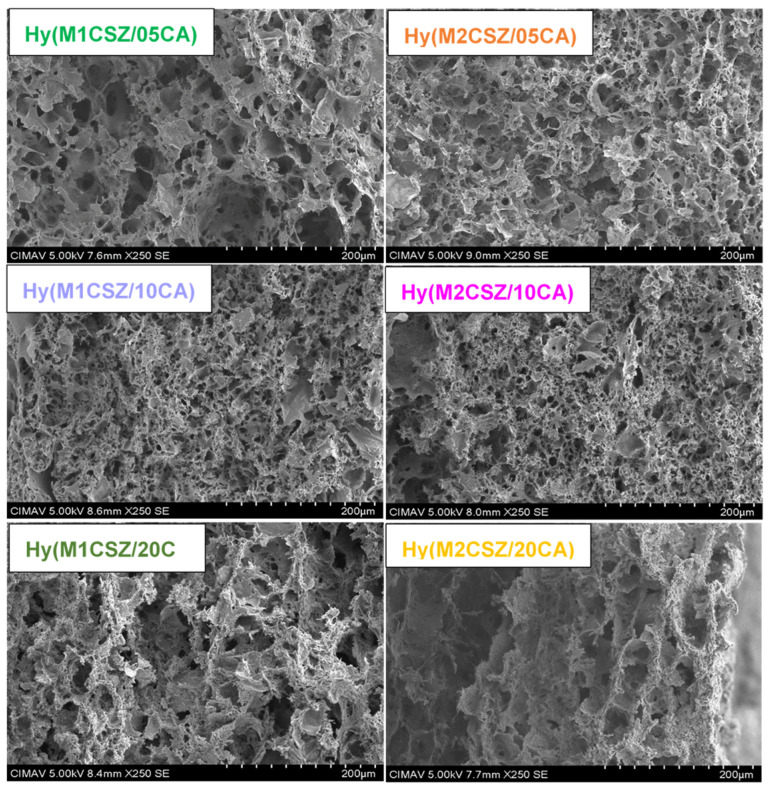
Scanning electron microscopy (SEM) micrographs of the synthesized hydrogels, showing surface morphology at 250× magnification.

Figure 4 presents the cross-sectional morphologies of the synthesized hydrogels, revealing a macroporous structure composed of well-defined porous cavities. The hydrogels Hy(M1CSZ/05CA) and Hy(M2CSZ/05CA), prepared with the lowest citric acid (CA) content, exhibit average pore sizes of 19.5 and 11.7 µm, respectively. However, increasing the CA concentration in the formulations Hy(M1CSZ/10CA), Hy(M1CSZ/20CA), Hy(M2CSZ/10CA), and Hy(M2CSZ/20CA) results in reduced pore sizes of 8.6, 8.7, 11.4, and 8.56 µm, respectively. This reduction can be attributed to a higher crosslinking density, which restricts polymer chain mobility and promotes the formation of a denser, more robust, and compact three-dimensional network. Notably, the presence of larger pores enables greater water absorption capacity, consistent with findings reported by Badra Pitaloka et al. [29].

### 2.4. Swelling Percentage

**Figure 5:**
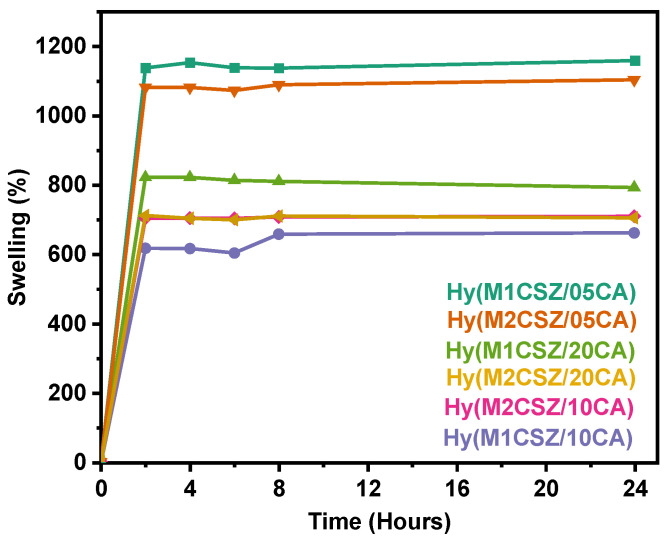
Swelling capacity of the synthesized hydrogels.

The swelling percentage of the hydrogels, evaluated over 24 h, reveals behavior characteristic of hydrophilic polymeric networks (Figure 5), with rapid water uptake during the first 2 h and equilibrium attainment before 4 h, remaining stable thereafter without syneresis or apparent degradation. An inverse relationship is observed between citric acid (CA) concentration and water absorption capacity: samples prepared with a 05CA/CSZ ratio exhibit the highest swelling values (>1100%) due to a more open network with lower crosslinking density, whereas increasing the CA content to 1.0 and 2.0 (mass ratio) significantly reduces swelling (600–800%). This reduced swelling is a consequence of greater physical restriction of polymer chains imposed by a higher density of ester linkages, which correlates directly with the increased intensity of the band at 1725 cm^−1^ observed in FTIR analysis. Regarding milling time, samples M1 (15 min) tend to show slightly higher swelling capacity than M2 (30 min), suggesting that prolonged milling may promote a more homogeneous distribution of the crosslinker and a slightly more compact network structure. Additionally, the rheological tests presented in Figure S1 (Supplementary Material) reveal a predominantly elastic (solid-like) behavior, characteristic of hydrogel networks [30]. This analysis focused on the hydrogels with the highest swelling degree, Hy(M1CSZ/05CA) and Hy(M2CSZ/05CA), using amplitude and frequency sweeps. In the amplitude sweep, both systems exhibited storage modulus (G′) values exceeding those of the loss modulus (G″) in the low-deformation range, confirming network stability.

To complement the hydrogel characterization, detailed analyses of pore size distribution, degree of substitution, and swelling kinetics are provided in the Supplementary Material.

### 2.5. Proposed Formation Pathway of Sargassum-Based Hydrogel

#### 2.5.1. Mechanochemical Activation and Amorphization

High-energy milling in a ball mill induces physical disintegration that reduces particle size to the micrometric scale [31]. This process promotes cellulose amorphization by disrupting the ordering of glucose chains and weakening intra and intermolecular hydrogen bonds, which drastically increases the surface area and accessibility of internal hydroxyl groups to chemical reagents [32]. Simultaneously, the biomass is intimately homogenized, creating a precursor matrix where the cellulosic fraction and alginates are exposed for subsequent reactions without the need for prior separation processes.

#### 2.5.2. Generalized Carboxymethylation of the Biopolymeric Matrix

During this process, etherification is not restricted to a single specific component; rather, the accessible hydroxyl groups distributed throughout the Sargassum biomass are carboxymethylated. This reaction converts previously insoluble structural polysaccharides into a highly anionic, water-soluble macromolecular network. While the native alginates, composed of mannuronic and guluronic acid blocks, largely maintain their stable polymeric backbone in the alkaline medium [33], their residual hydroxyl groups along with those of the newly modified biopolymeric fractions, act as active sites for subsequent crosslinking. The resulting product is a highly dispersed, reactive mixture of carboxymethylated biomass and native alginates.

#### 2.5.3. Thermal Crosslinking with Citric Acid via Cyclic Anhydride Intermediate

Citric acid acts as a crosslinking agent through a heat-activated esterification reaction. To achieve an absorption capacity close to 1160%, the citric acid/Sargassum mass ratio must be 0.5, as higher concentrations generate excessive crosslinking density that restricts network expansion and limits swelling [34,35].

#### 2.5.4. Origin of the Superabsorbent Capacity

**Figure 6:**
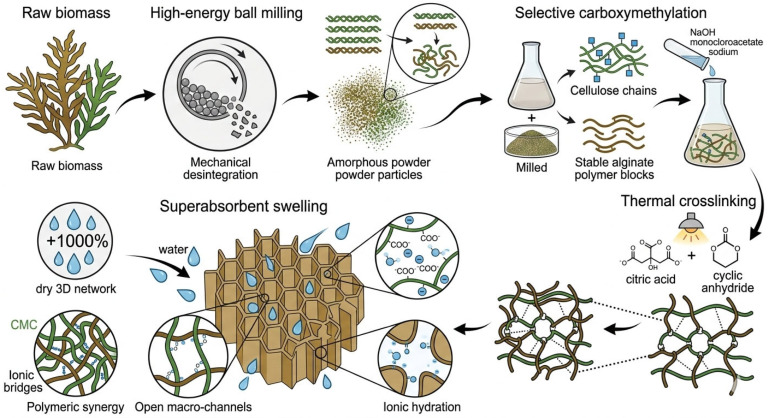
Schematic of the formulation of Sargassum-based hydrogel using citric acid.

This exceptional property is based on the synergy of three factors (Figure 6):

- Osmotic pressure and ionic hydration: The ionization of carboxylate groups (–COO^−^) from CMC and alginates generates a high density of negative charges, causing electrostatic repulsion between polymer chains and a strong osmotic pressure that drives water into the gel network.
- Macroporous structure: The material possesses an open pore morphology and microfibrillar channels that act as water reservoirs, enabling rapid diffusion and retention of large liquid volumes without structural collapse.
- Polymeric synergy: CMC provides high hydrophilicity, while alginates contribute to the formation of microdomains that stabilize the swollen network and enhance the hydrogel’s mechanical resistance through ionic interactions or hydrogen bonding.

## 3. Conclusions

This study demonstrates the feasibility of a simplified method for hydrogel synthesis through the direct conversion of Sargassum biomass into crosslinked carboxymethyl cellulose (CMC). This approach enables biomass processing without prior cellulose isolation steps, leveraging mechanochemical activation to generate CMC in situ while integrating native alginates as structural components of the polymeric matrix. Subsequent crosslinking with citric acid facilitated the formation of a three-dimensional network, the success of which was confirmed by FTIR spectroscopy through the appearance of characteristic ester linkage bands at 1725 cm^−1^ and 1232 cm^−1^. Swelling assays revealed that the absorption capacity is primarily governed by the crosslinking agent concentration. Specifically, a lower citric acid/carboxymethylated Sargassum ratio (0.5) yielded maximum swelling values of approximately 1160%, whereas higher ratios restricted network expansion to a range of 600–650%. While FTIR confirmed crosslink formation, a more rigorous quantitative assessment of the crosslinking degree is needed to precisely correlate it with network density. Additionally, long-term stability and cyclic swelling–deswelling evaluations fall outside the current scope but remain critical for assessing durability under repeated use. Despite these limitations, this study establishes a sustainable green chemistry pathway for Sargassum valorization, converting an ecological burden into a high-performance functional material by leveraging its native biochemical architecture.

## 4. Materials and Methods

All reagents used were of analytical grade (ACS, Washington, DC, USA): sodium hydroxide (NaOH; Macron Fine Chemicals, Phillipsburg, NJ, USA), isopropyl alcohol [(CH_3_)_2_CHOH], monochloroacetic acid (ClCH_2_COOH), citric acid monohydrate (C_6_H_8_O_7_·H_2_O), ethanol (C_2_H_5_OH), and acetone (CH_3_COCH_3_). These chemicals were primarily purchased from J.T. Baker (Phillipsburg, NJ, USA). The biological material consisted of brown macroalgae belonging to the genus *Sargassum*. Specimens were collected in August 2025 from Playa Delfines, Quintana Roo, Mexico. For sample preparation, seaweed was harvested at the tideline, manually cleaned to remove epiphytes and sand, and thoroughly rinsed with distilled water prior to use.

### 4.1. Methodology

The experimental procedure for hydrogel synthesis was structured into sequential stages to track the material’s evolution from cleaned biomass to its final functional form. During the mechanochemical processing stage, Sargassum (SZ) was subjected to high-energy mechanical activation via milling at two treatment times: 15 min (M1SZ) and 30 min (M2SZ). The milled biomass served as the substrate for carboxymethylation, yielding the corresponding carboxymethylated derivatives for each milling condition, designated as M1CSZ and M2CSZ, respectively. In the final synthesis stage, hydrogels were formed through chemical crosslinking using citric acid as the crosslinking agent. Systematic variations in crosslinker concentration enabled the evaluation of its effect on the physicochemical and swelling properties of the resulting polymer networks.

#### 4.1.1. Mechanochemical Activation: Preparation of Powdered Sargassum by Milling

**Figure 7:**
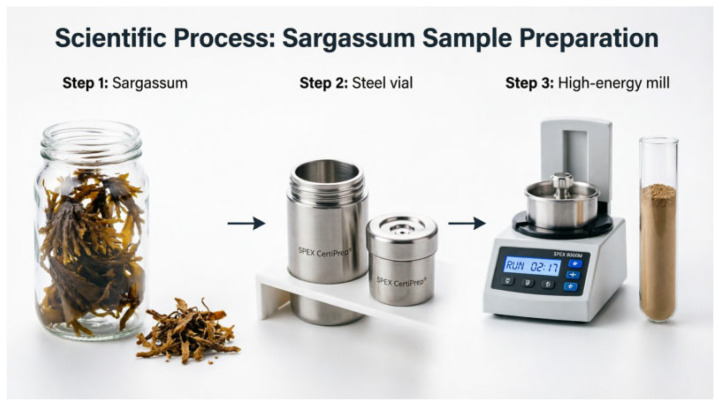
Schematic diagram of the mechanochemical activation illustrating the high-energy ball milling system (SPEX 8000M) used to transform Sargassum (SZ) into powders (M1SZ and M2SZ).

Sargassum (SZ) was subjected to high-energy milling in a SPEX 8000M high-energy ball mill equipped (HORIBA Instruments, Irvine, CA, USA) with six stainless steel balls: three with a 13 mm diameter (8.2 g each) and three with an 11 mm diameter (5.4 g each). For each milling run, the grinding vial was loaded with the six balls and 8 g of predried Sargassum (Figure 7). The system operates via high-frequency oscillatory motion that combines reciprocating displacements with short lateral movements at both ends of the vial, generating repetitive impacts and shear forces that promote particle size reduction and structural modification. Two milling durations were evaluated in a single cycle: 15 min and 30 min, yielding samples designated as M1SZ and M2SZ, respectively. Upon completion of the process, the recovered powder was sieved through a No. 40 mesh (425 µm).

#### 4.1.2. Chemical Functionalization: Carboxymethylation of Milled Sargassum

The carboxymethylation of Sargassum was carried out using an adaptation of the Williamson ether synthesis procedure described by He X. et al. [36]. Initially, 5 g of Sargassum powder were added to 133 mL of 2-propanol under vigorous stirring at room temperature until a homogeneous dispersion was achieved. Subsequently, 13 mL of a 30% (*w*/*v*) NaOH solution was added, and the mixture was stirred for 1 h. After this period, monochloroacetic acid (ClCH_2_COOH, 6 g) was slowly incorporated over 30 min, and the reaction was allowed to proceed for 3 h at 55 °C. Finally, the product was washed with 100 mL of an ethanol/water mixture (80:20, *v*/*v*) followed by acetone, filtered, and dried at 50 °C. The resulting samples were designated as M1CSZ and M2CSZ, corresponding to the 15 min and 30 min milled precursors, respectively.

#### 4.1.3. Final Product: Hydrogel Synthesis

Hydrogels were synthesized from carboxymethylated Sargassum (M1CSZ and M2CSZ) using citric acid (CA) as a crosslinking agent. Briefly, 1 g of carboxymethylated Sargassum (CSZ) was dispersed in 10 mL of distilled water at room temperature under constant stirring until a homogeneous viscous solution was obtained. Subsequently, citric acid was added at three different CA/CSZ mass ratios (0.5, 1.0, and 2.0), as detailed in Table 1. The resulting mixtures were poured into Petri dishes and subjected to thermal treatment in an oven at 80 °C for 15 h to induce crosslinking and hydrogel network formation [35]. The final products were designated with the prefix “Hy” followed by the corresponding crosslinker ratio.

### 4.2. Material Characterization

Hydrogels were characterized using a Shimadzu FTIR Affinity-1S spectrometer (Shimadzu Corporation, Tokyo, Japan), recording spectra in the range of 450–4000 cm^−1^. The longitudinal morphology of the hydrogels was examined by high-resolution imaging using a Hitachi SU3500 scanning electron microscope (SEM) (Hitachi High-Tech Corporation, Tokyo, Japan) operated in low-vacuum mode (60 Pa) at 15 kV. Prior to analysis, hydrogel samples were freeze-dried at −60 °C for 48 h. Dried hydrogel structures were sputter coated with gold and mounted on aluminum stubs using double sided conductive graphite tape. Thermogravimetric analysis (TGA) was performed on a TA Instruments SDT Q600 simultaneous thermal analyzer, (TA Instruments, New Castle, DE, USA), operating over a temperature range of 30–800 °C at a heating rate of 10 °C min^−1^ under nitrogen atmosphere. X-ray diffraction (XRD) patterns were acquired using a Bruker D8 Advance diffractometer (Bruker AXS, Karlsruhe, Germany), equipped with a Cu Kα radiation source (λ = 1.5406 Å). Rheological characterization of the viscoelastic hydrogels was carried out on a TA Instruments AR-G2 rotational rheometer (TA Instruments, New Castle, DE, USA). Hydrogel samples were cut into disks of 15 mm diameter and 1 mm thickness. Measurements were performed using a parallel-plate geometry (15 mm diameter) at 25 °C. Frequency sweep tests were conducted for each sample over a range of 0.1–100 Hz at a constant strain within the linear viscoelastic region.

### 4.3. Swelling Capacity Assay

The swelling capacity of the hydrogels was evaluated by immersion in distilled water at room temperature. Briefly, the initial dry mass of each hydrogel sample (*W_d_*) was recorded. Subsequently, samples were fully immersed in distilled water for 24 h to allow maximum liquid absorption. After this period, the hydrogels were carefully removed, excess surface water was gently blotted off using absorbent paper, and the swollen mass (*W_s_*) was immediately determined. The swelling percentage (% *S*) was calculated using the following equation [37]:

$$Percentage\;of\;swelling\;(\%\;S)=\frac{W_{s}\;-\;W_{d}}{W_{d}}\;\times \;100$$

The analysis was performed in triplicate, with the calculation of standard deviation.

## Figures and Tables

**Table 1:** Formulation parameters for hydrogel synthesis.

Hydrogel Nomenclature	CSZ (g/mL)	CA (g/mL)	Ratios CA/CSZ
Hy(M1CSZ/05CA)	0.10	0.05	0.5
Hy(M2CSZ/05CA)	0.10	0.05	0.5
Hy(M1CSZ/10CA)	0.10	0.10	1
Hy(M2CSZ/10CA)	0.10	0.10	1
Hy(M1CSZ/20CA)	0.10	0.20	2
Hy(M2CSZ/20CA)	0.10	0.20	2

Note: CSZ = carboxymethylated Sargassum; CA = citric acid crosslinker.

## Data Availability

Data are available upon reasonable request.

## Supplementary Materials

The following supporting information can be downloaded at: https://www.mdpi.com/article/10.3390/gels12050431/s1, Figure S1. Presents the amplitude (a) and frequency (b) sweeps for the hydrogels exhibiting the highest swelling percentages, Hy(M1CSZ/05CA) and Hy(M2CSZ/05CA); Figure S2. Comparison of the FTIR Spectra of sargassum (SZ), ground sargassum samples (M1SZ, M2SZ) and carboxymethylated derivatives (M1CSZ, M2CSZ); Figure S3. Pore size distribution of synthesized hydrogels: (a) Hy(M1CSZ/05CA), (b) Hy(M1CSZ/10CA), (c) Hy(M1CSZ/20CA), (d) (Hy(M2CSZ/05CA), (e) Hy(M2CSZ/10CA) and (f) Hy(M2CSZ/20CA); Table S1. Swelling rate parameters. References [10,30,38,39] are cited in the supplementary materials.
