# Supplementary material for: Superabsorbent Hydrogels Derived from Unpurified Sargassum Biomass via Direct Carboxymethylation and Crosslinking
Source: Gels. 2026 May 15;12(5):431. doi: 10.3390/gels12050431 (PMC13205862; doi:10.3390/gels12050431)
Supplement: Supplementary file 1 [file gels-12-00431-s001.zip › gels-4272136-supplementary.pdf]

Supplementary material

Article

Superabsorbent hydrogels derived from unpurified Sargassum biomass via direct carboxymethylation and crosslinking

C. Villalva-Cañavi, A.B. Jasso-Salcedo, D. Lardizabal-Gutierrez*

Table of contents

S1. Rheological tests

Figure S1. Presents the (a) amplitude and (b) frequency sweeps for the hydrogels exhibiting the highest swelling percentages, Hy(M1CSZ/05CA) and Hy(M2CSZ/05CA)

S2. FTIR spectra of sargassum (SZ), ground sargassum samples (M1SZ, M2SZ) and carboxymethylated derivatives (M1CSZ, M2CSZ).

Figure S2. Comparison of the FTIR Spectra of sargassum (SZ), ground sargassum samples (M1SZ, M2SZ) and carboxymethylated derivatives (M1CSZ, M2CSZ).

S3. Pore size distribution

Figure S3. Pore size distribution of synthesized hydrogels: (a) Hy(M1CSZ/05CA), (b) Hy(M1CSZ/10CA), (c) Hy(M1CSZ/20CA), (d) (Hy(M2CSZ/05CA), (e) Hy(M2CSZ/10CA) and (f) Hy(M2CSZ/20CA)

S4. Degree of substitution (DS)

S5. Swelling kinetics

Table S1. Swelling rate parameters

S1. Rheological tests

The rheological amplitude and frequency sweeps for the highly swelling hydrogels, Hy(M1CSZ/05CA) and Hy(M2CSZ/05CA), are shown in Figure S1. Within the low-strain regime, G′ remains significantly above G″ for both samples, indicating a dominant elastic character and validating the formation of a stable, solid-like polymeric network. [30] Figure 1 S (a). For the Hy(M1CSZ/05CA) hydrogel, the storage modulus (G′ ≈ 8.85 × 10^4 Pa) remains above the loss modulus (G″ ≈ 1.04 × 10^4 Pa) throughout the entire linear viscoelastic region (LVR), which extends up to approximately 0.3 % strain. In contrast, the Hy(M2CSZ/05CA) hydrogel exhibits a higher initial storage modulus (G′ ≈ 1.68 × 10^5 Pa; G″ ≈ 1.76 × 10^4 Pa) but a narrower LVR, limited to strain values near 0.1–0.2 %. The LVR limit was determined using the conventional criterion of a 10 % deviation from the initial G′ value, thereby defining the critical strain (γc). Beyond this point, both hydrogels show a progressive decrease in G′, indicating the onset of structural network disruption and the transition toward a nonlinear viscoelastic regime. The frequency sweep (0.1–100 Hz), shown in Figure S1 (b) reveals that for the Hy(M1CSZ/05CA) hydrogel, the storage modulus (G′) remains consistently above the loss modulus (G″) across the entire frequency range analyzed, with no crossover point observed. Both moduli exhibit weak frequency dependence, manifested as a slight increase in their values with increasing frequency. Similarly, the Hy(M2CSZ/05CA) hydrogel displays G′ values exceeding G″ throughout the evaluated range, along with a mild frequency dependence of both moduli. However, Hy(M2CSZ/05CA) exhibits higher G′ magnitudes compared to Hy(M1CSZ/05CA), suggesting a stiffer network structure. Collectively, the predominance of G′ over G″ and the low frequency dependence of both moduli are characteristic of three-dimensional networks with permanent crosslinks, confirming the typical behavior of chemically crosslinked hydrogels and their structural stability under dynamic perturbations.

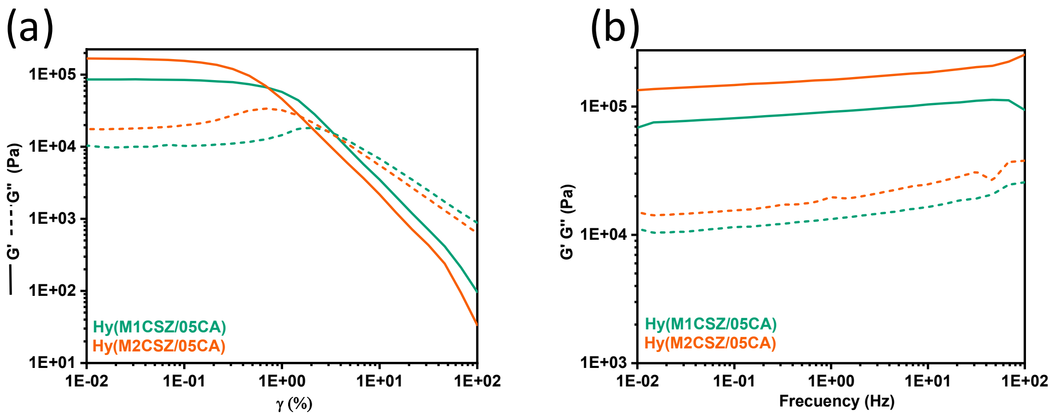

Figure S1. Presents the amplitude (a) and frequency (b) sweeps for the hydrogels exhibiting the highest swelling percentages, Hy(M1CSZ/05CA) and Hy(M2CSZ/05CA).

S2. FTIR spectra of sargassum (SZ), ground sargassum samples (M1SZ, M2SZ) and carboxymethylated derivatives (M1CSZ, M2CSZ).

Figure S2 presents a comparative FTIR analysis of raw SZ, milled samples (M1SZ, M2SZ), and carboxymethylated derivatives (M1CSZ, M2CSZ). Following carboxymethylation, an intensified band at 1322 cm^{-1} is observed, assigned to the symmetric stretching vibration of the C–O bond, which confirms the successful incorporation of carboxymethyl groups. Additionally, the band at 853 cm^{-1}, associated with the pyranose ring vibrations characteristic of algal polysaccharides, verifies the preservation of glycosidic linkages within the SZ structure after processing.

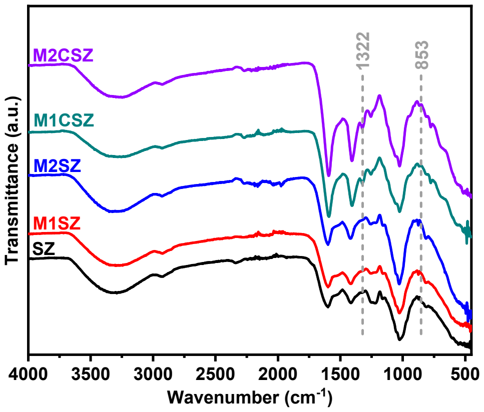

Figure S2. Comparison of the FTIR Spectra of sargassum (SZ), ground sargassum samples (M1SZ, M2SZ) and carboxymethylated derivatives (M1CSZ, M2CSZ).

S3. Pore size distribution

The pore analysis was conducted using Gatan Digital Micrograph software by processing the SEM images to obtain representative statistical measurements (Figure S3). An average of 75 pores per sample were evaluated to ensure the reliability of the results.

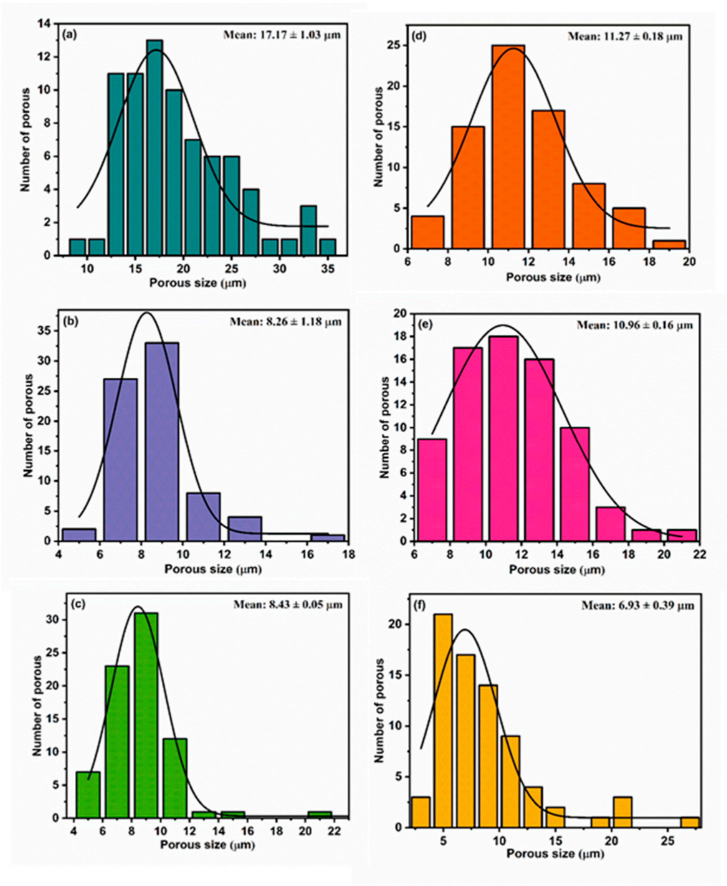

Figure S3. Pore size distribution of synthesized hydrogels: (a) Hy(M1CSZ/05CA), (b) Hy(M1CSZ/10CA), (c) Hy(M1CSZ/20CA), (d) (Hy(M2CSZ/05CA), (e) Hy(M2CSZ/10CA) and (f) Hy(M2CSZ/20CA)

S4. Degree of substitution (DS)

The DS was determined as follows: approximately 0.05 g of each sample was dissolved in 20 mL of 0.1 mol/L HCl solution under continuous stirring. The resulting solution was then titrated with 0.1 mol/L NaOH, and the volumes consumed to reach pH 2.1 (V1) and pH 4.3 (V2) were recorded. The carboxymethyl content (A, mmol/g) and DS were calculated using Equations (1) and (2):

$$A=(V_2-V_1) \times C/m \qquad (1)$$

DS = 0.203 x A/(1-0.058 x A) (2)

where V1 and V2 represent the NaOH volumes (mL) required to reach pH 2.1 and 4.3, respectively; C is the NaOH concentration (mol/L); and m is the sample mass (g).

The calculated DS values were 0.78 for M1CSZ and 0.70 for M2CSZ

These results are consistent with literature reports for carboxymethylated polysaccharides derived from plant biomass and macroalgae, which typically range between 0.4 and 1.0 for hydrogel applications (An et al., 2022)[10]. A DS within this interval ensures an optimal balance between hydrophilicity/swelling capacity and the availability of free hydroxyl groups for subsequent citric acid-mediated crosslinking.

S5. Swelling kinetic studies

The second-order swelling kinetics of the hydrogels were evaluated using the Schott's equation (Titilayo et al. (2012)[38], Seki et al. (2014)[39]:

$$t/W = 1/kW_{\infty}^{2} + t/W_{\infty}$$

where W represents the swelling degree of the hydrogel at time t, W_{∞} denotes the theoretical equilibrium swelling capacity, and k is the swelling rate constant. The W_{∞} and k were calculated from the slope of the plot t/W as function of t.

$$W_{\infty} = 1/\text{slope}$$

$$k = 1/(\text{Intercept} \times W_{\infty}^{2})$$

Table S1. Swelling rate parameters

Hydrogels	Parameter	Value
Hy(M1CSZ/05CA)	W_{∞} (%)	1111.11
	k (1/h)	0.01157
	R^2	0.99
Hy(M2CSZ/05CA)	W_{∞} (%)	1115.08
	k (1/h)	0.00810
	R^2	0.99

It is important to note that this kinetic analysis focused on the formulations with the most promising performance (Hy(M1CSZ/05CA) and Hy(M2CSZ/05CA)) to better understand the water absorption mechanisms of the hydrogel synthesized.

The kinetic parameters for hydrogels Hy(M1CSZ/05CA) and Hy(M2CSZ/05CA) are presented in Table S1, showing swelling rate constants of 0.01157 and 0.00810, respectively. The higher k value obtained for Hy(M1CSZ/05CA) indicates faster swelling kinetics, allowing the material to reach equilibrium in a shorter timeframe compared to Hy(M2CSZ/05CA).

Reference

30. Nadgorny M, Xiao Z, Connal LA. 2D and 3D-printing of self-healing gels: Design and extrusion of self-rolling objects. *Mol Syst Des Eng*. **2017** 1, 283–92. doi:10.1039/c7me00023e
10. An, Y., Liu, H., Li, X., Liu, J., Chen, L., Jin, X., Chen, T., Wang, W., Liu, Z., Zhang, M., & Liu, F.. Carboxymethylation modification, characterization, antioxidant activity and anti-UVC ability of Sargassum fusiforme polysaccharide. *Carbohydrate Research*, **2022**, 515. https://doi.org/10.1016/j.carres.2022.108555
38. J Titilayo Bamgbose, A Adesina Bamigbade, E O Dare, A A Lasisi, A Njah Equilibrium swelling and kinetic studies of highly swollen chitosan film. *J. Chem. Chem. Eng*., **2012**, 6.
39. Y Seki, A Altinisik, B Demircioğlu, C Tetik Carboxymethylcellulose (CMC)-hydroxyethylcellulose (HEC) based hydrogels: *Synthesis and characterization. Cellulose*, **2014**, 21, 1689-1698. DOI 10.1007/s10570-014-0204-8
